# Performance of non‐invasive prenatal testing in vanishing‐twin and multiple pregnancies: results of TRIDENT‐2 study

**DOI:** 10.1002/uog.70015

**Published:** 2025-09-06

**Authors:** J. C. A. van Eekhout, C. J. Bax, L. van Prooyen Schuurman, E. C. Becking, A. J. E. M. van der Ven, D. Van Opstal, E. M. J. Boon, M. V. E. Macville, M. N. Bekker, R. J. H. Galjaard, the Dutch NIPT Consortium, E.A. Sistermans, L. Henneman, A. Polstra, E. Voorhoeve, S.L. Zelderen‐Bhola, E.M.J. Boon, M.P.R. Lombardi, M.C. Bakker, E.J. Bradley, E.M.J. Boon, C. Louwerens‐Zintel, M. Smit, M.C. van Maarle, M.B. Tan‐Sindhunata, K. van der Meij, H. Meij, C.J. Bax, E. Pajkrt, I.H. Linskens, L. Martin, J.T. Gitsels‐van der Wal, R. J. H. Galjaard, D. van Opstal, M.I. Srebniak, F.M. Sarquis Jehee, I.H.I.M. Hollink, F. Sleutels, W. de Valk, W.H. Deelen, A.M.S. Joosten, K.E.M. Diderich, M.E. Redeker, A.T.J.I. Go, M.F.C.M. Knapen, S. Galjaard, A.K.E. Prinsen, A.P.G. Braat, M.J.V. Hoffer, N.S. den Hollander, E.J.T. Verweij, M.C. Haak, M.V.E. Macville, S.J.C. Stevens, A. van der Wijngaard, L.H. Houben, M.A.A. van Esch‐Lennarts, L. Hamers, A.G.P. Jetten, S.A.I. Ghesquiere, B. de Koning, M. Zamani Esteki, C.J. Heesterbeek, C.E.M. de Die‐Smulders, H. Brunner, M.J. Pieters, A.B.C. Coumans, D.F.C.M. Smeets, B.H.W. Faas, D. Westra, M.M. Weiss, I. Derks‐Prinsen, I. Feenstra, M. van Rij, E. Sikkel, R.F. Suijkerbuijk, B. Sikkema‐Raddatz, I.M. van Langen, K. Bouman, L.K. Duin, G.H. Schuring‐Blom, K.D. Lichtenbelt, M.N. Bekker, E. van Vliet‐Lachotzki, J. Pot, A. J. E. M. van der Ven, S van 't Padje

**Affiliations:** ^1^ Department of Clinical Genetics, Erasmus MC University Medical Centre Rotterdam The Netherlands; ^2^ Department of Obstetrics and Gynecology, Amsterdam UMC University of Amsterdam Amsterdam The Netherlands; ^3^ Department of Obstetrics and Gynecology University Medical Center Utrecht, Utrecht University Utrecht The Netherlands; ^4^ Verloskundigenpraktijk Velp Velp The Netherlands; ^5^ Department of Human Genetics Amsterdam UMC Amsterdam The Netherlands; ^6^ Department of Clinical Genetics, GROW School for Oncology and Reproduction Maastricht University Medical Centre Maastricht The Netherlands

**Keywords:** cell‐free DNA, multiple pregnancy, non‐invasive prenatal testing, prenatal screening, vanishing‐twin pregnancy

## Abstract

**Objective:**

To evaluate the performance of non‐invasive prenatal testing (NIPT) in vanishing‐twin and multiple pregnancies.

**Methods:**

This study was conducted as part of the TRIDENT‐2 study, in which NIPT was offered as a first‐tier screening test to women with a multiple pregnancy or vanishing‐twin pregnancy between 1 June 2020 and 31 March 2023 in The Netherlands. Abnormal NIPT results were investigated by follow‐up invasive prenatal testing and/or postnatal genetic testing. Chorionicity, amnionicity and type of vanishing twin were determined on first‐trimester ultrasound examination. A vanishing twin was classified as Type I in the presence of an additional empty gestational sac or as Type II when an additional non‐viable embryo was observed on ultrasound. The performance of NIPT (sensitivity, specificity and positive predictive value (PPV)) was assessed.

**Results:**

NIPT was performed in 655 women with a vanishing‐twin pregnancy, of which 231 were Type I, 374 were Type II and 50 were of unknown type. Women with a Type‐II vanishing‐twin pregnancy had a significantly higher likelihood of receiving an abnormal NIPT result compared to those with Type I (12.6% *vs* 1.7%; *P* < 0.001). Among the 655 vanishing twins, NIPT was indicative of trisomies 21, 18 and 13 in 17 cases (screen‐positive rate (SPR), 2.60%), four cases (SPR, 0.61%) and eight cases (SPR, 1.22%), respectively. NIPT was indicative of additional findings (chromosomal aberrations other than the major trisomies) in 29 cases that underwent genome‐wide NIPT (SPR, 6.16%). In 7/17 (41.2%) cases, trisomy 21 was confirmed in the remaining fetus by cytogenetic follow‐up. The sensitivity of NIPT for the detection of trisomy 21 in vanishing‐twin pregnancies was 100% (95% CI, 59.0–100%), the specificity was 98.5% (95% CI, 97.2–99.3%) and the PPV was 41.2% (95% CI, 18.4–67.1%). None of the cases of trisomy 18 (*n* = 4), trisomy 13 (*n* = 8) or with additional findings (*n* = 29) were confirmed in the remaining fetus. Of the 12 cases in which NIPT was performed after 15 weeks' gestation, there were no discordant‐positive results. NIPT was performed in 2992 women with a dichorionic diamniotic twin pregnancy, 1112 women with a monochorionic twin pregnancy and 75 women with a triplet pregnancy. Of the 2992 dichorionic twin pregnancies, 27 NIPT results were indicative of trisomy 21, 18 or 13 (SPR, 0.90%), of which 21 were confirmed in one fetus. In addition, 16 NIPT results were indicative of an additional finding (SPR, 0.75%), of which three were confirmed by invasive prenatal testing. In 3/1112 (0.3%) monochorionic twin pregnancies, NIPT was indicative of trisomy 21, which was confirmed in both fetuses in all cases. In addition, NIPT was indicative of an additional finding in four cases (SPR, 0.49%), of which none were confirmed. Of the 75 triplet pregnancies, NIPT was indicative of trisomy 21 in one case (SPR, 1.33%); trisomy 21 was confirmed in one of the three triplet fetuses.

**Conclusions:**

Women with a Type‐II vanishing‐twin pregnancy are more likely to receive an abnormal NIPT result compared to those with a Type‐I vanishing‐twin pregnancy. NIPT appears suitable for detecting trisomy 21 in the remaining fetus of a vanishing‐twin pregnancy, however, none of the trisomy 18, trisomy 13 or additional findings could be confirmed on cytogenetic follow‐up. There were no discordant‐positive results reported when NIPT was conducted after 15 weeks' gestation. © 2025 The Author(s). *Ultrasound in Obstetrics & Gynecology* published by John Wiley & Sons Ltd on behalf of International Society of Ultrasound in Obstetrics and Gynecology.

## INTRODUCTION

Non‐invasive prenatal testing (NIPT) has revolutionized prenatal screening by analyzing total cell‐free DNA (cfDNA), which contains both maternal and placental/fetal DNA, in maternal blood to detect chromosomal abnormalities in singleton pregnancies^1^, ^2^, ^3^, ^4^. However, data on the performance of NIPT in vanishing‐twin pregnancies remain limited, making interpretation of NIPT results difficult. A vanishing twin is defined as the spontaneous reduction of one or more fetuses in a multiple pregnancy during the first trimester^5^, ^6^, with reported incidence rates ranging from 0.6–36%^7^, ^8^. Two types of vanishing twin have been described: vanishing twin with an additional empty gestational sac visible on ultrasound, and that with an additional gestational sac containing a non‐viable embryo^9^. Since chromosomal aberrations are a major cause of first‐trimester pregnancy loss, analysis of cfDNA from the deceased fetus using NIPT could result in a discordant‐positive result^10^, ^11^, ^12^, ^13^, ^14^. In multiple pregnancies, cfDNA from the placentae of both fetuses is shed into the maternal bloodstream, potentially complicating analysis. While trisomy 21 detection is well studied, data on the detection of trisomies 18 and 13 and additional findings remain limited^15^, ^16^.

Over the years, first‐trimester combined testing (FTCT), involving ultrasound and testing of first‐trimester serum markers, has been the conventional screening strategy for identifying pregnancies with an increased risk of trisomies 21, 18 or 13. In singleton and multiple pregnancies, the detection rate (DR) of FTCT for trisomy 21 is about 85–90%, with a false‐positive rate of 5%^17^, ^18^. The accuracy of FTCT in vanishing‐twin pregnancies, however, is unknown. In contrast, NIPT offers a DR of around 98% for trisomy 21 in singleton pregnancies, with a false‐positive rate below 1%^1^, ^2^. As a result, Belgium and The Netherlands, two countries which offer NIPT as a first‐tier screening test, have largely phased out FTCT. In The Netherlands, FTCT has been discontinued since October 2021.

Given the limited data on the accuracy of NIPT in vanishing‐twin pregnancies and the more extensive but still incomplete data on its accuracy for chromosomes other than chromosome 21 in multiple pregnancies, essential information for comprehensive pretest counseling on test characteristics is lacking. Therefore, our objective was to assess the performance of NIPT in vanishing‐twin and multiple pregnancies.

## METHODS

This study was part of the Trial by Dutch Laboratories for Evaluation of Non‐Invasive Prenatal Testing, phase 2 (TRIDENT‐2) study. The TRIDENT‐2 study is a nationwide implementation study of NIPT in the Dutch prenatal screening program for trisomies 21, 18 and 13. Since the start of the TRIDENT‐2 study in April 2017, NIPT has been offered exclusively for singleton and monochorionic twin pregnancies. As of June 2020, NIPT has also been available for dichorionic twin pregnancies, multiple pregnancies and vanishing‐twin pregnancies. The current study presents the results for monochorionic and dichorionic twin pregnancies, multiple pregnancies and vanishing‐twin pregnancies registered between 1 June 2020 and 31 March 2023 (the results for singleton pregnancies have been published previously^1^, ^19^). Women who opted for NIPT could opt for reporting of chromosomes 21, 18 and 13 exclusively, or reporting that included additional findings, i.e. all large chromosomal aberrations (> 10–20 Mb) in addition to the major trisomies. Sex chromosomes were not analyzed, as this is not part of the government license. In addition, NIPT is not able to detect triploidy in The Netherlands. Pregnant women paid an out‐of‐pocket fee of €175 (180 USD) for NIPT as part of the TRIDENT‐2 study^20^. High‐risk pregnancies, classified based on medical history but not maternal age alone, were not included. The aim of the TRIDENT‐2 study was to assess the performance of NIPT in the general obstetric population. Further information regarding the inclusion and exclusion criteria of the TRIDENT‐2 study has been published previously^1^.

For multiple pregnancies, baseline characteristics and pregnancy outcome information were obtained from medical records and the national Dutch prenatal screening database (Peridos; Coöperatie Landelijk Bureau Prenatale Screening, Utrecht, The Netherlands) in cases of an abnormal NIPT result. For vanishing‐twin pregnancies, this information was collected in both abnormal and normal NIPT cases, as it was required to confirm the presence of a vanishing twin. Information regarding chorionicity, amnionicity and type of vanishing twin was determined at the first‐trimester ultrasound examination. We distinguish two types of vanishing twin: Type I has an additional empty gestational sac visible on ultrasound, and Type II has an additional gestational sac containing a non‐viable embryo. The viability ultrasound was performed between 7 + 0 and 10 + 0 weeks' gestation to confirm viability, identify multiple pregnancy and rule out ectopic pregnancy. The dating ultrasound was conducted between 10 + 0 and 12 + 6 weeks to determine the expected due date of the pregnancy. The first‐trimester anomaly scan (offered in a research setting since 1 September 2021^21^) was performed between 12 + 3 and 14 + 3 weeks to screen for major structural abnormalities in the fetus, such as open skull or thick nuchal translucency. The second‐trimester anomaly scan was conducted between 19 + 0 and 21 + 0 weeks to screen for structural abnormalities, assess fetal growth and determine the position of the placenta. In the case of a Type‐I vanishing twin, timing of fetal demise was estimated at 5 weeks, because in most normal pregnancies an embryo is detectable after 6 weeks^22^. For cases of Type‐II vanishing twin, the timing of fetal demise was estimated using the crown–rump length (CRL) formula of Robinson and Fleming^23^ if the CRL was reported for the dead twin. If the CRL was not reported, it was not possible to estimate the timing of fetal demise.

Maternal peripheral blood samples were collected after 11 weeks and sent to one of the three clinical genetic laboratories (Amsterdam UMC (location VUMC), Rotterdam Erasmus MC and Maastricht UMC+, The Netherlands) designated to perform NIPT as part of the TRIDENT‐2 study. Methods of cfDNA extraction and analysis are described in a previously published paper^19^ and in Appendix S2. The combined fetal fraction was measured for both twins using the VeriSeq NIPT Solution v2 software (Illumina Netherlands, Eindhoven, The Netherlands), as detailed in Appendix S2.

Additional findings were divided into three groups: rare autosomal trisomies, defined as trisomies other than trisomies 21, 18 and 13; structural chromosomal aberrations, defined as a single gain and/or loss of a large chromosomal segment; and complex profiles, defined as multiple gains and/or losses of whole chromosomes or chromosomal segments.

In the case of an abnormal NIPT result, women were referred to one of the seven regional centers for prenatal diagnosis, for genetic counseling and, if desired, genomic testing during the pregnancy (chorionic villus sampling, amniocentesis and/or maternal blood) and an anomaly scan. The type of diagnostic follow‐up test depended on the type of chromosomal aberration, gestational age, patient preference and the presence or absence of ultrasound abnormalities. In the case of a vanishing twin, only the remaining viable fetus was tested. Chorionic villus, amniotic fluid and maternal blood cells were analyzed using genomic arrays, fluorescent *in‐situ* hybridization and/or conventional karyotyping. Postnatal genetic testing was performed mostly on umbilical cord blood or tissue or on skin biopsies.

Definitions of NIPT test results were as follows: true positive, defined as a high‐risk NIPT result confirmed by (invasive) genomic testing of the developing fetus or by phenotyping; discordant positive, defined as a high‐risk NIPT result unconfirmed by (invasive) genomic testing of the developing fetus or by phenotyping; discordant negative, defined as a low‐risk NIPT result after which a chromosomal aberration was detected by (invasive) pre‐ or postnatal genomic testing; and true negative, defined as a low‐risk NIPT result with no abnormal genotyping or phenotyping of the developing fetus or the infant after birth. As part of the TRIDENT‐2 protocol, all discordant‐negative results were reported to the project leader. Since all prenatal and postnatal cytogenetic testing in The Netherlands is performed in one of the seven university medical centers involved in this study, the chance of missing a discordant‐negative result was extremely low.

Descriptive statistics were utilized to describe maternal age, body mass index, gestational age and birth weight. Categorical data were compared using the chi‐square test or Fisher's exact test, depending on the outcome frequency. The performance of NIPT (sensitivity, specificity and positive predictive value (PPV)) was calculated using a 2 × 2 table. Statistical testing was conducted using R software version 4.2.1 (R Project for Statistical Computing, Vienna, Austria). *P* < 0.05 was considered statistically significant.

TRIDENT‐2 was licensed by the Minister of Health, Welfare, and Sport (license number: 1017420‐153371‐PG). All women signed an informed consent form for use of their data for research purposes.

## RESULTS

### Vanishing‐twin pregnancies

**Figure 1:**
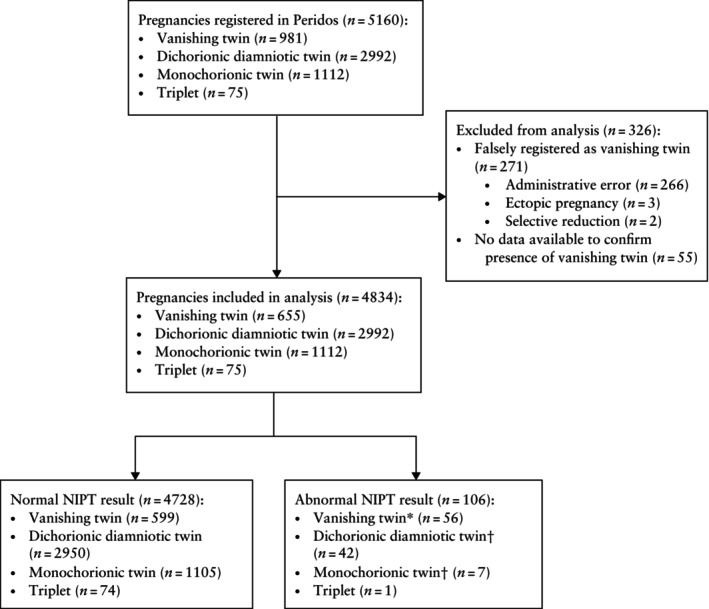
Flowchart showing inclusion of pregnancies in study that underwent non‐invasive prenatal testing (NIPT). *Including two cases in which two chromosomal aberrations were detected. †Including one case in which two chromosomal aberrations were detected.

Between 1 June 2020 and 31 March 2023, 981 vanishing‐twin pregnancies were registered in Peridos. After data collection, 271 (27.6%) cases were excluded from the analysis because they were determined to be falsely registered as a vanishing‐twin pregnancy (administrative error (*n* = 266), ectopic pregnancy (*n* = 3) and selective reduction (*n* = 2)), and 55 (5.6%) cases were excluded because there were no data available to confirm the presence of a vanishing twin (Figure 1). In total, NIPT was performed in 655 women with a vanishing‐twin pregnancy. The majority of pregnant women who opted for NIPT chose genome‐wide analysis (471/655 (71.9%)).

Table 1 presents the characteristics of women with a vanishing‐twin pregnancy, according to abnormal or normal NIPT result. Women with an abnormal NIPT result were significantly older than women with a normal NIPT result (median, 37.0 *vs* 33.0 years; *P* < 0.001); this difference remained when considering only additional findings detected using NIPT (median, 36.3 *vs* 32.6 years; *P* < 0.001). Of the 655 vanishing‐twin pregnancies, 231 were Type I, 374 were Type II and 50 were unknown. Characteristics according to type of vanishing twin are presented in Table 2. Women with a Type‐II vanishing twin had an increased likelihood of receiving an abnormal NIPT result compared to those with Type I (12.6% *vs* 1.7%; *P* < 0.001). Details of fetal chromosome aberrations detected using NIPT in vanishing‐twin pregnancies and results of follow‐up investigations are given in Table S1.

Of the 655 vanishing‐twin pregnancies, 56 received an abnormal NIPT result, including two cases in which two chromosomal aberrations were detected (29 major trisomies: screen‐positive rate (SPR), 4.43%; 29 additional findings: SPR, 6.16%). These aberrations were considered separately when determining the accuracy of the NIPT results. NIPT results were indicative of trisomy 21 in 17 cases (SPR, 2.60%), including one case in which NIPT also indicated trisomy 13. Cytogenetic follow‐up testing in the remaining twin was performed in 15/17 cases of trisomy 21. In the two cases in which no follow‐up testing was conducted, a normal phenotype was confirmed after birth. In total, 7/17 (41.2%) cases of trisomy 21 were confirmed in the remaining twin by cytogenetic follow‐up. The performance of NIPT for trisomy 21 in the remaining fetus in vanishing‐twin pregnancies is presented in Table 3. NIPT was indicative of trisomy 18 in four cases (SPR, 0.61%) and of trisomy 13 in eight cases (SPR, 1.22%). Among the trisomy 13 cases, one case also demonstrated trisomy 21, as mentioned above, whereas another exhibited both trisomy 13 and trisomy 7. In all cases of trisomy 18 and in 5/8 cases of trisomy 13, pre‐ and/or postnatal follow‐up testing was performed but no trisomies could be confirmed in the remaining twin. In the three cases of trisomy 13 that did not undergo follow‐up testing, a normal phenotype was confirmed after birth. The number of trisomy 18 and trisomy 13 cases was too small to be able to reliably calculate the performance of NIPT. NIPT results were indicative of additional findings in 29 of the vanishing‐twin pregnancies that underwent genome‐wide NIPT (SPR, 6.16%). One case each of trisomy 9, 10, 12, 14 and 20, three cases of trisomy 7 (one of which also indicated trisomy 13, as mentioned above), three cases of trisomy 22, eight cases of trisomy 16 and 10 cases of trisomy 15 were detected. In 25 of the cases with additional findings, pre‐ and/or postnatal follow‐up testing was performed in the remaining twin and none of the additional findings could be confirmed. In the four cases that did not undergo follow‐up testing, a normal phenotype was confirmed after birth. In three cases (one each of trisomies 7, 15 and 18), the placenta of the remaining fetus was examined postpartum but none of the chromosomal aberrations could be confirmed. No discordant‐negative cases were reported for any of the major trisomies.

**Table 1:** Maternal and baseline characteristics of women with a vanishing‐twin pregnancy, according to abnormal or normal non‐invasive prenatal testing (NIPT) result

Characteristic	Abnormal NIPT result (*n* = 56)	Normal NIPT result (*n* = 599)	*P*
Maternal age (years)	37.0 (34.0–39.0)	33.0 (30.0–35.0)	< 0.001
Maternal BMI (kg/m^2^)	23.6 (21.4–24.9)†	23.9 (21.7–27.2)†	0.085
Spontaneous conception	32/50 (64.0)	243/335 (72.5)	0.305
Nulliparous	18/55 (32.7)	255/509 (50.1)	0.021
Vanishing twin			< 0.001
Type I	4/51 (7.8)	227/554 (41.0)	
Type II	47/51 (92.2)	327/554 (59.0)	
GA at fetal demise (weeks)	8 + 0 (6 + 3 to 8 + 5)‡	5 + 0 (5 + 0 to 6 + 5)§	< 0.001
GA at NIPT (weeks)	12 + 0 (11 + 2 to 12 + 4)	12 + 0 (11 + 2 to 12 + 5)	0.802
Method of NIPT			0.005
Targeted	7 (12.5)	177 (29.5)	
Genome‐wide	49 (87.5)	422 (70.5)	
Interval between fetal demise and NIPT (weeks)	4 + 2 (3 + 1 to 5 + 4)‡	6 + 3 (5 + 3 to 7 + 2)§	< 0.001
Vanishing twin visible on viability ultrasound*	25/45 (55.6)	347/471 (73.7)	0.027
Vanishing twin visible on dating ultrasound*	42/45 (93.3)	331/464 (71.3)	0.003

Data are given as median (interquartile range), *n*/*N* (%) or *n* (%).

* Indicates whether vanishing twin was observed, not first instance of diagnosis; in some cases, vanishing twin was documented in medical records but not explicitly mentioned in the ultrasound report.

† Missing data for one pregnancy.

‡ Missing data for 13 pregnancies.

§ Missing data for 183 pregnancies. BMI, body mass index; GA, gestational age.

**Table 2:** Maternal and baseline characteristics of women with a vanishing‐twin pregnancy, according to type of vanishing twin

Characteristics	Type‐I vanishing twin (*n* = 231)	Type‐II vanishing twin (*n* = 374)	*P*
Maternal age (years)	37.0 (34.0–39.0)	33.0 (30.0–35.0)	< 0.001
Maternal BMI (kg/m^2^)	23.4 (21.3–26.4)*	24.2 (21.9–27.3)*	0.043
Spontaneous conception	104/133 (78.2)	158/225 (70.2)	0.128
Nulliparous	91/201 (45.3)	160/324 (49.4)	0.409
GA at fetal demise (weeks)	5 + 0 (5 + 0 to 5 + 0)	7 + 0 (6 + 1 to 8 + 3)†	< 0.001
GA at NIPT (weeks)	11 + 6 (11 + 2 to 12 + 4)	12 + 0 (11 + 4 to 12 + 6)	0.061
Method of NIPT			0.308
Targeted	72 (31.2)	101 (27.0)	
Genome‐wide	159 (68.8)	273 (73.0)	
Interval between fetal demise and NIPT (weeks)	6 + 6 (6 + 2 to 7 + 4)	4 + 5 (3 + 4 to 6 + 0)†	< 0.001
Abnormal NIPT result	4 (1.7)	47 (12.6)	< 0.001

Data are given as median (interquartile range), *n*/*N* (%) or *n* (%).

* Missing data for one pregnancy.

† Missing data for 146 pregnancies. BMI, body mass index; GA, gestational age; NIPT, non‐invasive prenatal testing.

**Table 3:** Performance of non‐invasive prenatal testing for detection of trisomy 21 (T21) in women with a vanishing twin or dichorionic diamniotic (DCDA) twin pregnancy

Type of pregnancy	High‐risk T21 result	True‐positive result	Discordant‐positive result	Discordant‐ negative result	True‐negative result	Sensitivity (95% CI) (%)	Specificity (95% CI) (%)	PPV (95% CI) (%)
Vanishing	17/655	7/17	10/17	0/17	638/638	100	98.5	41.2
twin	(2.6)	(41.2)	(58.8)	(0)	(100)	(59.0–100)	(97.2–99.3)	(18.4–67.1)*
DCDA	21/2992	21/21	0/21	0/21	2971/2971	100	100	100
twin	(0.7)	(100)	(0)	(0)	(100)	(83.9–100)	(99.9–100)	(83.9–100)

Data are given as *n*/*N* (%), unless stated otherwise.

* Positive predictive value (PPV) calculated for remaining fetus.

**Figure 2:**
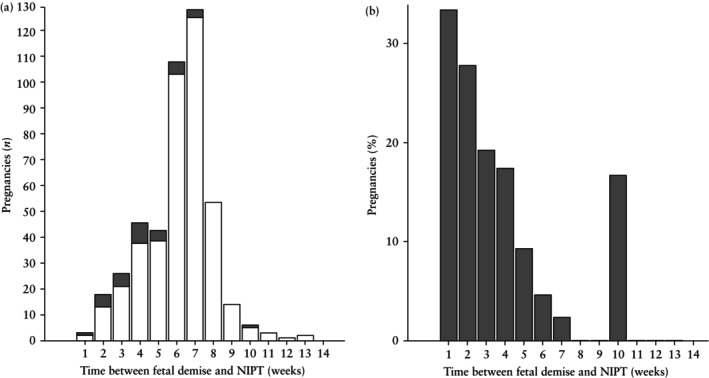
Distribution of concordant (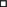) and discordant‐positive (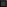) non‐invasive prenatal testing (NIPT) results (a) and percentage of NIPT results that were discordant (b), per weekly interval between fetal demise and NIPT in vanishing‐twin pregnancies.

In 228/374 (61.0%) Type‐II vanishing‐twin pregnancies, we were able to estimate the time of fetal demise (median, 7 + 0 (interquartile range (IQR), 6 + 1 to 8 + 3) weeks). For all Type‐I vanishing‐twin pregnancies, the estimated time of fetal demise was 5 + 0 weeks. The median gestational age at fetal demise was 8 + 0 weeks in pregnancies with a discordant‐positive NIPT result and 5 + 0 weeks for those with a concordant (true positive or true negative) NIPT result (*P* < 0.001). When considering only Type‐II vanishing twins, the difference in gestational age at fetal demise between those with a discordant‐positive result and those with a concordant result was smaller (7 + 6 weeks *vs* 7 + 1 weeks), but remained significant (*P* = 0.010). There was a significant difference in the interval between fetal demise and NIPT between pregnancies with a discordant‐positive result and those with a concordant result (4 + 1 weeks *vs* 6 + 3 weeks; *P* < 0.001). Figure 2a shows the total number of vanishing‐twin pregnancies with a discordant‐positive and those with a concordant NIPT result per weekly increment in the interval between fetal demise and NIPT. Figure 2b shows the percentage of women with a discordant‐positive NIPT result per weekly increment in the interval between fetal demise and NIPT. Notably, as the interval increased, the percentage of women with a discordant‐positive NIPT result declined, with the exception of one discordant‐positive case in which NIPT was conducted 10 weeks after fetal demise (1/6 (16.7%)). When comparing targeted and genome‐wide NIPT analysis, we observed this same weekly decline (Figure S1). However, in one case with a Type‐I vanishing twin, NIPT was conducted 10 weeks following fetal demise and indicated trisomy 16, which could not be confirmed in the remaining fetus by amniocentesis. This fetus showed signs of fetal growth restriction and was delivered with a birth weight < 3^rd^ percentile.

**Figure 3:**
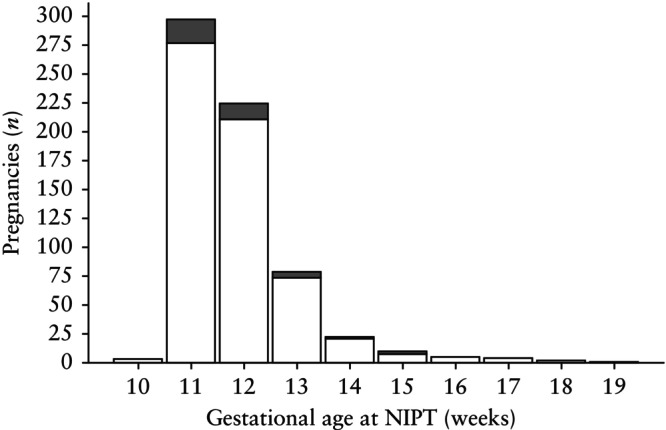
Distribution of concordant (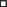) and discordant‐positive (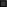) non‐invasive prenatal testing (NIPT) results per gestational week in vanishing‐twin pregnancies.

The distribution of the number of discordant‐positive and concordant NIPT results according to each weekly increment of gestational age at the time of NIPT is shown in Figure 3. After 15 weeks, our study revealed no discordant‐positive NIPT results; a total of 12 tests were performed during this time. Before 15 weeks, 636 tests were performed, of which 42 had discordant‐positive results. For the remaining seven pregnancies, follow‐up testing was not performed so it was not possible to classify the NIPT result as concordant or discordant. The difference between discordant‐positive results in those that underwent NIPT ≤ 15 weeks *vs* > 15 weeks was not statistically significant (*P* = 1.0). This finding was the same when considering targeted and genome‐wide NIPT analysis separately (Figure S2).

### Multiple pregnancies

Between 1 June 2020 and 31 March 2023, a total of 4179 women with a multiple pregnancy (2992 dichorionic diamniotic (DCDA) twins, 1112 monochorionic twins and 75 triplets) were included in the TRIDENT‐2 study for NIPT. The median maternal age of women with a multiple pregnancy who had an abnormal NIPT result was 34.0 (IQR, 32.0–37.0) years, which is higher than that of women who had a normal NIPT result (median, 32.0 (IQR, 30.0–35.0) years). The median gestational age at NIPT was 11 + 6 (IQR, 11 + 2 to 12 + 2) weeks. The majority of pregnant women who opted for NIPT chose genome‐wide analysis (3020/4179 (72.3%)). Among the 50 multiple pregnancies with an abnormal NIPT result, 42 (84.0%) were DCDA, six (12.0%) were monochorionic diamniotic, one (2.0%) was monochorionic monoamniotic and one (2.0%) was a trichorionic triamniotic triplet pregnancy. Details of fetal chromosomal aberrations detected by NIPT in multiple pregnancies and the results of follow‐up investigations are given in Table S2.

### Dichorionic diamniotic twin pregnancies

Of the 2992 DCDA twin gestations, NIPT was indicative of trisomy 21 in 21 cases (SPR, 0.70%). In one case, concurrent trisomy 22 was detected on NIPT. Cytogenetic follow‐up testing was performed in 20/21 cases of trisomy 21. The one case without confirmatory testing involved intrauterine fetal demise (IUFD) of one fetus, which had shown trisomy 21 features on ultrasound, such as increased nuchal translucency, generalized edema and absent nasal bone. In all cases in which follow‐up testing was performed, trisomy 21 was confirmed in one of the two fetuses. The performance of NIPT for trisomy 21 in DCDA twins is presented in Table 3. NIPT was indicative of trisomy 18 in one case (SPR, 0.03%), which was confirmed by cytogenetic follow‐up. NIPT was indicative of trisomy 13 in five cases (SPR, 0.17%). Cytogenetic follow‐up testing was performed in 3/5 cases of trisomy 13, of which none were confirmed. However, in one of these cases only the surviving fetus was tested for trisomy 13 following IUFD of the other twin, which was presumed to be affected by trisomy 13 owing to increased nuchal translucency, hydrops fetalis and fetal growth restriction; trisomy 13 was not confirmed by cytogenetic follow‐up in the surviving twin. In one case that did not undergo confirmatory testing, a normal phenotype was confirmed after birth in both twins. In the other case, one fetus had trisomy 13 features on ultrasound and experienced IUFD, while a normal phenotype was confirmed in the surviving twin at birth.

NIPT was indicative of additional findings in 16/2141 DCDA pregnancies that underwent genome‐wide NIPT (SPR, 0.75%). One case each of trisomy 2, 3, 6, 8 and 22 were detected, with the case of trisomy 22 also showing concurrent trisomy 21 as mentioned above. Additionally, seven cases of trisomy 7, three cases with a structural aberration (gain 1q, loss 7q and loss 20q) and one case with a complex NIPT profile were reported. The cases of trisomy 2, trisomy 8 and gain 1q were confirmed by invasive prenatal testing. The woman whose pregnancy had a complex NIPT profile was diagnosed with Hodgkin lymphoma. No discordant‐negative cases were reported for any of the major trisomies.

After follow‐up testing, selective fetal reduction was performed in 18/35 (51.4%) DCDA pregnancies with an abnormal NIPT result that had not already ended in IUFD. Among these selective reductions, 15 cases were due to confirmed fetal trisomy 21, one was due to fetal trisomy 18, one was due to fetal mosaic trisomy 2 and one was due to multiple congenital abnormalities observed on ultrasound, consistent with arthrogryposis multiplex congenita, a monogenetic disease beyond the scope of NIPT. In six DCDA pregnancies, IUFD occurred in one of the twins. In two cases, IUFD was associated with a possible trisomy 13, which was not confirmed as invasive testing was not performed because of IUFD. In three cases, IUFD was associated with trisomy 21, which was confirmed in two cases and assumed in one case. In one DCDA pregnancy, both fetuses died; NIPT was indicative of both trisomy 21 and trisomy 22 in this pregnancy. Trisomy 21 was confirmed in one of the fetuses, while trisomy 22 could not be confirmed by amniocentesis. The second fetus exhibited severe early‐onset fetal growth restriction.

### Monochorionic twin and triplet pregnancies

In the group of 1112 monochorionic twins, NIPT was indicative of trisomy 21 in three cases (SPR, 0.27%). In one of these three cases, NIPT was also indicative of trisomy 13 (SPR, 0.09%). All cases of trisomy 21 were confirmed in both fetuses during or after pregnancy, but the trisomy 13 was not. NIPT was indicative of additional findings in 4/824 monochorionic twins that underwent genome‐wide NIPT (SPR, 0.49%). None of the three structural aberrations (gain 7p, gain 18p and loss 21q) or the trisomy 10 were confirmed in either of the twin fetuses. Of the 75 triplet pregnancies, NIPT was indicative of trisomy 21 in one case (SPR, 1.33%); trisomy 21 was confirmed in one of the three triplet fetuses.

## DISCUSSION

This study shows that NIPT can accurately predict the presence of trisomy 21 in multiple pregnancies and appears to be suitable for detecting trisomy 21 in the remaining fetus of a vanishing‐twin pregnancy. Additionally, we found that women with a Type‐II vanishing‐twin pregnancy are 7‐times more likely to receive an abnormal NIPT result than those with a Type‐I vanishing‐twin pregnancy (12.6% *vs* 1.7%), possibly owing to earlier fetal demise in a Type‐I vanishing twin, resulting in lower fetal cfDNA levels^24^.

The SPR of NIPT in vanishing‐twin pregnancies observed in the present study is considerably higher than that reported for singleton pregnancies in previously published data of the TRIDENT‐2 study^1^ (4.43% *vs* 0.48% for the major trisomies; 6.16% *vs* 0.36% for additional findings). This aligns with findings of other studies and is presumably due to chromosomal aberrations in the deceased fetus^25^. Nevertheless, NIPT appears suitable for detecting trisomy 21 in the remaining fetus of a vanishing‐twin pregnancy. Similar conclusions were made in the study of Kleinfinger *et al*.^14^, which reported a PPV of 50% (95% CI, 21–79%) for trisomy 21, while the studies of van Riel *et al*.^12^ and Zou *et al*.^13^ reported lower PPVs of 19% (95% CI, 4–46%) and 11% (95% CI, 3–48%), respectively. Although broadly overlapping 95% CIs prevent us from establishing statistical significance for these differences, variations in PPV could also reflect differences in study populations (low‐risk^12^ and mixed^14^ (one of which included only pregnancies conceived via assisted reproductive technology^13^)), NIPT technology and the timing of the studies.

Trisomy 21 is more often confirmed in the remaining fetus than trisomies 18 and 13. This is most likely because trisomy 21 is more viable than trisomies 18 and 13, increasing the likelihood of detection in the remaining fetus^26^, ^27^, ^28^. Moreover, trisomy 18 and especially trisomy 13 are more often involved in confined placental mosaicism (CPM) than is trisomy 21^29^. In such cases, only the placenta is affected by the chromosomal aberration while the fetus is chromosomally normal, which is the case in around 50% of NIPT results indicating trisomy 13 in singleton pregnancies.

Although fetal cfDNA levels decline rapidly after delivery^30^, they can remain detectable for several weeks following fetal demise in a vanishing‐twin pregnancy^31^, ^32^, ^33^, ^34^, ^35^. Our study found a weekly decline in the number of discordant‐positive cases, with only one case detected more than 8 weeks after fetal demise, possibly owing to CPM. However, because the timing of fetal demise is only an estimate, assessing gestational age at the time of NIPT provides a more reliable measurement. We observed no discordant‐positive results after 15 weeks' gestation, a finding that is consistent with those of two previous studies^11^, ^13^. However, it is important to acknowledge that the total number of NIPT performed after 15 weeks in our study was limited.

Our findings can be utilized in the counseling of women with a vanishing‐twin pregnancy. NIPT can be performed at around 10–11 weeks, but carries an increased likelihood of obtaining an abnormal result. If the result is abnormal, in addition to offering invasive prenatal testing, which is the gold standard, repeating NIPT after 15 weeks can be considered an alternative. Another option is to delay NIPT until 15 weeks, which decreases the likelihood of receiving an abnormal result. Delaying NIPT also minimizes the period of uncertainty, as amniocentesis is often performed from 16 weeks, allowing for prompt intervention if necessary. The approach should be individualized based on the preference of the patient.

In our study, no discordant‐negative results were identified. This is promising, given the assumed risk of missed chromosomal abnormalities in DCDA twins. In such pregnancies, both placentae contribute to the total amount of fetal cfDNA. Since fetal aneuploidy, especially trisomies 18 and 13 (but not trisomy 21), is associated with a lower fetal fraction, a higher contribution from the normal twin may obscure the lower fetal fraction of the affected fetus, potentially resulting in a discordant‐negative case^36^, ^37^, ^38^, ^39^. This study showed that NIPT accurately predicts the presence of trisomy 21 in low‐risk women with a multiple pregnancy. However, the number of trisomy 18 and trisomy 13 cases was too small to calculate the test performance reliably. This is consistent with the findings of other studies, making it challenging to assess the performance of NIPT for detecting trisomies 18 and 13^12^, ^14^, ^15^, ^16^.

The main strength of this study is the large population of vanishing‐twin pregnancies and the distinction between types of vanishing twin. Nevertheless, our study has several limitations. First, NIPT was not repeated to assess at which point the fetal cfDNA of the vanishing twin became undetectable. Second, sex chromosomes were not analyzed, as this is not covered by the government license. Therefore, monosomy X, a common cause of fetal demise, could not be detected and accounted for. In addition, because of the nature of the NIPT that was used (the read count method), it was not possible to identify triploidy, another cause of spontaneous fetal demise^40^. In countries in which sex chromosomes and triploidy are analyzed, there may be more discordant‐positive results among cases of vanishing twin.

In conclusion, women with a Type‐II vanishing‐twin pregnancy are 7‐times more likely to have an abnormal NIPT result than those with a Type‐I vanishing‐twin pregnancy. NIPT appears suitable for detecting trisomy 21 in the remaining fetus of a vanishing‐twin pregnancy, however, none of the trisomy 18, trisomy 13 or additional findings could be confirmed on cytogenetic follow‐up. There were no discordant‐positive results reported when NIPT was conducted > 15 weeks' gestation. Future research should focus on the timing of the disappearance of fetal cfDNA of the vanishing twin to determine the optimal gestational age at which to perform NIPT in vanishing‐twin pregnancies. In multiple pregnancies, NIPT accurately predicted the presence of trisomy 21. The sample size was too small to calculate the performance of NIPT for the detection of other chromosomal aberrations.

## Supporting information


**Appendix S1** Dutch NIPT Consortium members.

**Appendix S2** Methods for cell‐free DNA extraction and analysis.

**Table S1** Details of fetal chromosomal aberrations detected by non‐invasive prenatal testing (NIPT) in vanishing‐twin pregnancies and results of follow‐up investigations.

**Table S2** Details of fetal chromosomal aberrations detected by non‐invasive prenatal testing (NIPT) in multiple pregnancies and results of follow‐up investigations.

**Figure S1** Impact of time from fetal demise to non‐invasive prenatal testing (NIPT) on result concordance in vanishing‐twin pregnancies, according to targeted (a,b) or genome‐wide (c,d) NIPT.

**Figure S2** Relationship between gestational age at non‐invasive prenatal testing (NIPT) and result concordance in vanishing‐twin pregnancies, in those that underwent targeted (a) and genome‐wide (b) NIPT.

## Data Availability

The data that support the findings of this study are available from the corresponding author upon reasonable request.
