# Supplementary material for: Performance of non‐invasive prenatal testing in vanishing‐twin and multiple pregnancies: results of TRIDENT‐2 study
Source: Ultrasound Obstet Gynecol. 2025 Sep 6;66(6):738–46. doi: 10.1002/uog.70015 (PMC12671934; doi:10.1002/uog.70015)
Supplement: Supplementary file 1 — Appendix S1 Dutch NIPT Consortium members. [file UOG-66-738-s005.docx]

**The Dutch NIPT Consortium (Date version: March 24, 2022)**

The Dutch NIPT Consortium consists of obstetric care givers, laboratory specialists, and other professionals from:

E.A. Sistermans^1^

L. Henneman^1^

A. Polstra^1^

E. Voorhoeve^1^

S.L. Zelderen-Bhola^1^

E.M.J. Boon^1^

M.P.R. Lombardi^1^

M.C. Bakker^1^

E.J. Bradley^1^

E.M.J. Boon^1^

C. Louwerens-Zintel^1^

M. Smit^1^

M.C. van Maarle^1^

M.B. Tan-Sindhunata^1^

K. van der Meij^1^

H. Meij^1^

C.J. Bax^2^

E. Pajkrt^2^

I.H. Linskens^2^

L. Martin^3^

J.T. Gitsels-van der Wal^3^

R. J. H. Galjaard^4^

D. van Opstal^4^

M.I. Srebniak^4^

F.M. Sarquis Jehee^4^

I.H.I.M. Hollink^4^

F. Sleutels^4^

W. de Valk^4^

W.H. Deelen^4^

A.M.S. Joosten^4^

K.E.M. Diderich^4^

M.E. Redeker^4^

A.T.J.I. Go^5^

M.F.C.M. Knapen^5^

S. Galjaard^5^

A.K.E. Prinsen^5^

A.P.G. Braat^6^

M.J.V. Hoffer^7^

N.S. den Hollander^7^

E.J.T. Verweij^8^

M.C. Haak^8^

M.V.E. Macville^9^

S.J.C. Stevens^9^

A. van der Wijngaard^9^

L.H. Houben^9^

M.A.A. van Esch-Lennarts^9^

L. Hamers^9^

A.G.P. Jetten^9^

S.A.I. Ghesquiere^9^

B. de Koning^9^

M. Zamani Esteki^9^

C.J. Heesterbeek^9^

C.E.M. de Die-Smulders^9^

H. Brunner^9^

M.J. Pieters^10^

A.B.C. Coumans^10^

D.F.C.M. Smeets

B.H.W. Faas^11^

D. Westra^11^

M.M. Weiss^11^

I. Derks-Prinsen^11^

I. Feenstra^11^

M. van Rij^11^

E. Sikkel^12^

R.F. Suijkerbuijk^13^

B. Sikkema-Raddatz^13^

I.M. van Langen^13^

K. Bouman^13^

L.K. Duin^14^

G.H. Schuring-Blom^15^

K.D. Lichtenbelt^15^

M.N. Bekker^16^

E. van Vliet-Lachotzki^17^

J. Pot^18^

A. J. E. M. van der Ven^19^

S van ‘t Padje

^1^ Department of Human Genetics, Amsterdam UMC, University of Amsterdam, Amsterdam, the Netherlands

^2^ Department of Obstetrics and Gynecology, Amsterdam UMC, University of Amsterdam, Amsterdam, the Netherlands

^3^ Department of Midwifery Science, AVAG and the EMGO Institute for Health and Care Research, VU University Medical Center, Amsterdam, Netherlands

^4^ Department of Clincial Genetics, Erasmus MC, University Medical Centre, Rotterdam, The Netherlands

^5^ Department of Obstetrics and Gynecology, Erasmus MC, University Medical Centre, Rotterdam, The Netherlands

^6^ Department of Information and Technology, Erasmus MC, University Medical Centre, Rotterdam, The Netherlands

^7^ Department of Clinical Genetics, Leiden University Medical Center, Leiden, the Netherlands

^8^ Department of Obstetrics and Gynecology, Division of Fetal Therapy, Leiden University Medical Center, Leiden, the Netherlands

^9^ Department of Clinical Genetics, Maastricht University Medical Centre, Maastricht, The Netherlands

^10^ Department of Obstetrics and Gynecology, Maastricht University Medical Centre, Maastricht, The Netherlands

^11^ Department of Clinical Genetics, Radboud University Medical Center, Nijmegen, the Netherlands

^12^ Department of Obstetrics and Gynecology, Radboud University Medical Center, Nijmegen, the Netherlands

^13^ Department of Clinical Genetics, University Medical Centre Groningen, University of Groningen, Groningen, the Netherlands

^14^ Department of Obstetrics and Gynecology, University Medical Centre Groningen, University of Groningen, Groningen, the Netherlands

^15^ Department of Clinical Genetics, University Medical Center Utrecht, Utrecht University, Utrecht, The Netherlands

^16^ Department of Obstetrics and Gynecology, University Medical Center Utrecht, Utrecht University, Utrecht, The Netherlands

^17^ Dutch Genetic Alliance, VSOP, Soest, the Netherlands

^18^ Erfocentrum, Amersfoort, The Netherlands

^19^ Verloskundigenpraktijk Velp, Velp, The Netherlands
