# Supplementary material for: Performance of non‐invasive prenatal testing in vanishing‐twin and multiple pregnancies: results of TRIDENT‐2 study
Source: Ultrasound Obstet Gynecol. 2025 Sep 6;66(6):738–46. doi: 10.1002/uog.70015 (PMC12671934; doi:10.1002/uog.70015)
Supplement: Supplementary file 2 — Appendix S2 Methods for cell‐free DNA extraction and analysis. [file UOG-66-738-s004.docx]

**Appendix 2: Methods for cell-free DNA extraction and analysis**

The VeriSeq NIPT Solution v.1.0.9 en v2.1.0, were used according to the specifications of the supplier (Illumina), which involves cfDNA extraction, library preparation, consecutive 36-bp paired-end sequencing on a NextSeq500 and corporate bioinformatic NIPT analysis. In parallel, bioinformatic NIPT analysis was performed using the WISECONDOR (v.2.0.1) algorithm with a resolution of approximately 10–15 Mb at the sequencing depth used. Individual z-scores per 1 Mbp bin size and Stouffer’s Z score over multiple 1 Mbp bins were calculated. The Z score cut-off of 3 was employed for calling trisomies and subchromosomal aberrations based on the sliding window approach.

The combined fetal fraction (FF) was measured for both twins with the VeriSeq NIPT Solution v2 software. The VeriSeq NIPT Assay Software uses information from both the cfDNA fragment size distribution and the differences in genomic coverage between maternal and fetal cfDNA to calculate a FF estimate. For twin pregnancies this is a combined FF. A minimum cut-off for solely fetal fraction is not set. As a quality metric an individual Fetal Aneuploidy Confidence Test (iFACT) is performed. This QC metric combines FF estimation with run metrics associated with coverage to determine whether the system has statistical confidence to either make a call on a given sample (PASS) or not (FAIL). For aneuploidy detection, a log likelihood ratio (LLR) is computed for each sample by taking into account coverage-based scores and the estimated FF for a given sample. The LLR is the probability of a sample being affected (i.e., an aneuploidy is detected) given the observed coverage and FF versus the probability of a sample being unaffected given the same observed coverage. When a sample is flagged by the user as a Twin, the assumptions about the FF of the sample are changed. For a Twin sample, the FF is assumed to represent both fetuses equally. Thus, the LLR computation takes into account the coverage-based scores and half of the estimated FF for that sample. This assumption about estimated FF in Twin samples is only used in the LLR calculations and is not used in iFact cutoffs or in the reported FF estimates. The conservative assumption of reduced FF in the LLR calculations for twin samples is intended to mitigate missed aneuploidy calls in these samples.

GRCh37 was used as reference genome. In case the parents opted for targeted testing of trisomy 21, 18, and 13, VeriSeq was performed in ‘basic’ mode and for WISECONDOR a filter was applied to reveal only the results of chromosomes 21, 18, and 13 and while masking other autosomes before the results were made available for interpretation. Chromosome 19 analysis by WISECONDOR is not reliable, because of a shortage of reference bins, and is therefore excluded from analysis. Sex chromosomes were not analyzed
