# Supplementary figures and images for: Performance of non‐invasive prenatal testing in vanishing‐twin and multiple pregnancies: results of TRIDENT‐2 study
Source: Ultrasound Obstet Gynecol. 2025 Sep 6;66(6):738–46. doi: 10.1002/uog.70015 (PMC12671934; doi:10.1002/uog.70015)

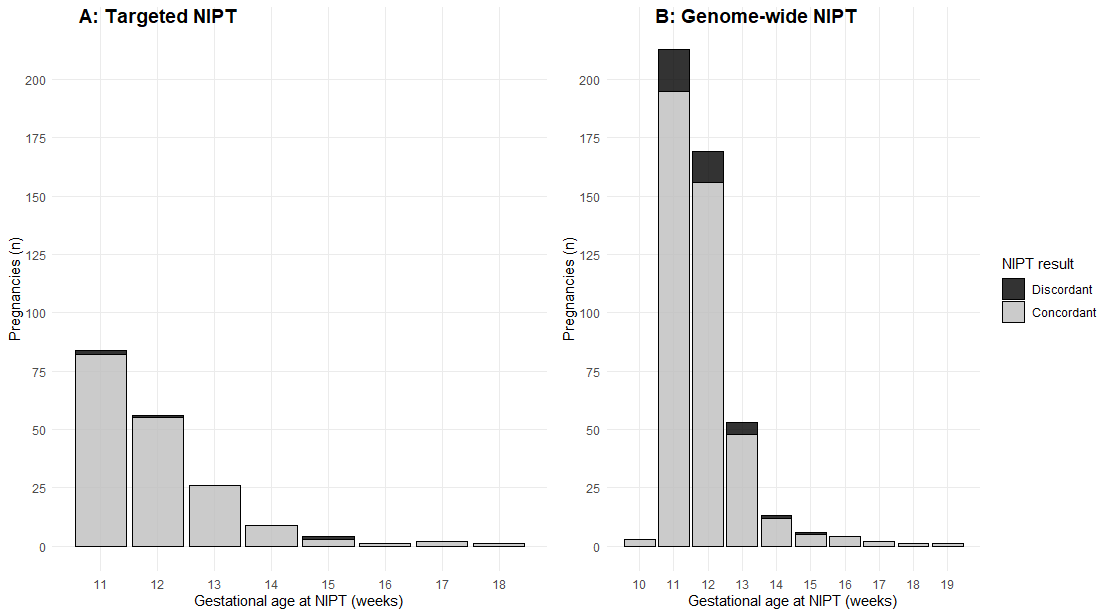

Supplement: Supplementary file 6 — Figure S2 Relationship between gestational age at non‐invasive prenatal testing (NIPT) and result concordance in vanishing‐twin pregnancies, in those that underwent targeted (a) and genome‐wide (b) NIPT. [file UOG-66-738-s003.png]
